# Automated assessment of regional muscle volume and hypertrophy using MRI

**DOI:** 10.1038/s41598-020-59267-x

**Published:** 2020-02-10

**Authors:** Mirko Mandić, Eric Rullman, Per Widholm, Mats Lilja, Olof Dahlqvist Leinhard, Thomas Gustafsson, Tommy R. Lundberg

**Affiliations:** 1 Division of Clinical Physiology, Department of Laboratory Medicine, Karolinska Institutet, and Unit of Clinical Physiology, Karolinska University Hospital, Stockholm, Sweden; 2 Cardiovascular Theme, Karolinska Institutet, Karolinska University Hospital Huddinge, Stockholm, Sweden; 3 AMRA Medical AB, Linköping, Sweden; 4 Department of Radiology, and Department of Medical and Health Sciences, Linköping University, Linköping, Sweden

**Keywords:** Computational biophysics, Translational research

## Abstract

This study aimed to validate a fully automatic method to quantify knee-extensor muscle volume and exercise-induced hypertrophy. By using a magnetic resonance imaging-based fat-water separated two-point Dixon sequence, the agreement between automated and manual segmentation of a specific ~15-cm region (partial volume) of the quadriceps muscle was assessed. We then explored the sensitivity of the automated technique to detect changes in both complete and partial quadriceps volume in response to 8 weeks of resistance training in 26 healthy men and women. There was a very strong correlation (r = 0.98, P < 0.0001) between the manual and automated method for assessing partial quadriceps volume, yet the volume was 9.6% greater with automated compared with manual analysis (P < 0.0001, 95% limits of agreement −93.3 ± 137.8 cm^3^). Partial muscle volume showed a 6.0 ± 5.0% (manual) and 4.8 ± 8.3% (automated) increase with training (P < 0.0001). Similarly, the complete quadriceps increased 5.1 ± 5.5% with training (P < 0.0001). The intramuscular fat proportion decreased (P < 0.001) from 4.1% to 3.9% after training. In conclusion, the automated method showed excellent correlation with manual segmentation and could detect clinically relevant magnitudes of exercise-induced muscle hypertrophy. This method could have broad application to accurately measure muscle mass in sports or to monitor clinical conditions associated with muscle wasting and fat infiltration.

## Introduction

Body composition plays a crucial role for overall health but also in specific sports and clinical settings. A number of pathological conditions are associated with reduced muscle mass, including muscular dystrophies, spinal cord injuries, sarcopenia, cancer, heart failure, neurological disease and inflammatory myopathies. In sports, muscle mass can be a major determinant of performance, and athletic injuries are typically associated with reduced muscle mass^1^. In all of the scenarios where muscle atrophy is evident, loss of strength and functional capacity most often occur in parallel^2^.

Numerous methods and techniques have been developed to assess body composition. These techniques include hydro-densitometry, air-displacement plethysmography, dual-energy x-ray absorptiometry (DXA), ultrasound and bio-impedance. In particular, the use of the DXA-method has increased in popularity over recent years, and is now commonly used in research studies assessing lean mass in cross-sectional or interventional studies^3,4^. While the DXA-method is relatively easy to use and requires essentially no manual data analysis, it is still subject to a number of limitations when detailed measurements of regional muscle mass are warranted^4^. The DXA scan uses ionizing radiation, provides only 2-dimensional projections of the body, and is based on several assumptions regarding segment constancy in tissue composition^5^. Moreover, the DXA-method underestimates the degree of age-related loss of muscle mass^6^, and it is not possible to separate between different muscle groups, or to quantify muscle or fat content within specific regions of muscle groups. For more detailed and accurate analysis of single muscle groups, therefore, more detailed imaging techniques such as computed tomography (CT) or magnetic resonance imaging (MRI) should be considered.

Since its clinical introduction, the MRI has revolutionized the diagnosis and treatment of various pathological conditions. Image acquisition time, image quality and advanced functional and anatomic imaging have all been optimized as a result of improvements in magnetic resonance techniques, pulse sequence acquisition strategies and novel hardware components^7^. Despite these advances, when using MRI to assess changes in muscle size with various interventions, post processing of images is still time-consuming and most often relies on manual segmentation where muscle boundaries are traced image by image for the subsequent calculation of muscle cross-sectional area or volume. Although manual segmentation is generally reproducible^8^, the time-consuming analysis limits its use in large-scale studies. Furthermore, inter-observer variability could be an issue^9^, and there are challenges in defining tissue boundaries or in separating individual muscles.

Recently, semi-automated and automated volume quantification techniques using threshold differences in pixels/voxels between muscle and other tissues have been developed. However, these techniques might not allow for volumetric analysis^10^, and there are still cases where semi-automated techniques rely on manual input, which increases the risk for inter-observer variability and makes them only moderately less time-consuming^10–12^. Currently, the most detailed automated segmentation techniques allow for separation of major anatomical compartments in whole body MR images based on two-point Dixon imaging techniques^13^. These water-fat separated images enable high soft-tissue contrast, allowing for detailed measurements of the muscle and fat volumes. This technique has recently been used to develop a fully automated segmentation technique requiring minimal manual input^13^. In short, the method is separated in four different steps starting with water-fat separated imaging with correction for intensity inhomogeneity followed by non-rigid registration of multiple atlases to the acquired image target. Muscle tissue is then classified using a voting scheme based on the multiple atlases registered. The last step is to quantify the muscle tissue volume based on the tissue classification and the local fat signal^14^. Despite this major advancement and the report of excellent test-retest reliability and correlation with manual segmentation^13,15,16^, the method has not yet been developed to distinguish between specific muscle groups of the lower limbs. This is important in the sports science setting where interventions often target specific muscles that are vital for performance in a specific context. Thus, to our knowledge, no automated technique has been validated to accurately quantify the volume of specific regions within a muscle group, and to detect relevant training-induced hypertrophy.

To this background, the overall goal of this study was to develop the automated segmentation technique to quantify muscle volume of the quadriceps muscle group of the anterior thigh. Specifically, we addressed the agreement between automated and manual segmentation of a specific ~15 cm region of the quadriceps muscle and explored the sensitivity of the automated technique to detect small but meaningful changes in whole-muscle volume in response to an 8-week resistance training intervention in young healthy individuals.

## Methods

### General design

The MRI images were obtained from subjects taking part in a randomized clinical trial recently published^17^. Young healthy men and women undertook a supervised resistance-training program targeting the knee extensor muscles for 8 weeks. Before and after the training program, MRI images were acquired using two different set-ups, one for the manual segmentation, and one for the automated technique developed by AMRA Medical AB (Linköping, Sweden).

### Subjects

While 31 subjects completed the clinical trial, the data set in this study is based on 26 subjects, 13 women and 13 men (age 26.6 ± 4.5, body mass 74.6 ± 16.1 kg, height 170.6 ± 10.6 cm), since five subjects were excluded due to geometrical distortions in the images to be processed with the automated technique (see further explanation below). Subjects were considered as recreationally active individuals, not conducting structured resistance or endurance training on a regular basis. Subjects were randomized to receiving daily doses of either 1200 mg ibuprofen (n = 15) or 75 mg acetylsalicylic acid (n = 16) for the entire study duration. Since the drug-intervention was irrelevant for the purpose of the current study, we focus on the averaged training response across the entire cohort. The study was approved by the Regional Ethical Review board in Stockholm. All research was performed in accordance with relevant guidelines/regulations, and informed consent was obtained from all participants prior to the start of the study.

### Training program

The training program is described in detail elsewhere^17^. In brief, the intervention consisted of 20 sessions scheduled 2–3 times per week for 8 weeks. The exercise was targeting the m. quadriceps and was performed unilaterally on two different knee extensor devices (regular weight-stack and flywheel device). Each of the subject’s leg was assigned to a specific device during the whole intervention. Each session started with a standardized warm-up before the participants performed 4 × 7 maximal repetitions in the flywheel machine (YoYo Technology Inc., Stockholm, Sweden) followed by 4 × 8–12 repetitions to failure in the weight stack machine (Worlds Class, Stockholm, Sweden). The starting order of the machines was altered in between each training session.

### MRI scans

**Figure 1:**
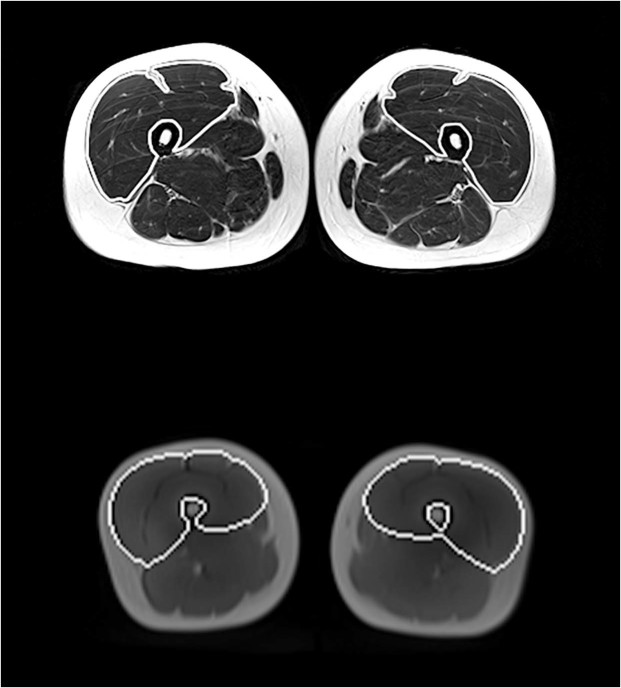
Transverse/axial sections of the manual (upper panel) and automated (lower panel) segmentation techniques. The white lines represent a visual clarification of the two methods displaying how the muscle boundaries of the knee extensors have been traced at the same anatomical location (mid-thigh). It should be noted that the lines have been added for visual clarification of the region of interest and they do not represent the actual tracing. However, the segmentation line for the automated method has been placed in full agreement with the actual tracing captured in a 2-dimensional screenshot.

Two different sequences of MRIs were run during the same occasion, before and after the training period (Fig. 1). In order to minimize the influence of fluid shift on muscle volume, subjects rested in the supine position for 1 h prior to any scan^18^. A custom-made foot-restrain device ensured a fixed-limb position and that there was no compression of thigh muscles. Preliminary localization (scout) images were obtained to confirm identical positioning across pre- and post-scans. To ensure that the same segment was scanned before and after training, the top of the caput femoris was used as an anatomical landmark when setting the field of view (FOV) for each scan. In both MRI sequences, cross-sectional images in the axial plane were obtained using a 1.5-Tesla Siemens Magnetom Aera unit (Siemens Healthcare, Germany).

In the T2-weighted Turbo Spin Echo-sequence used for the manual analysis, the acquisition parameters were: TE 110 ms, TR 5723 ms, number of signal averages; 3, FOV 48.5 (x-direction) × 39.4 (y-direction cm, voxel size 0.95 × 0.95 × 10 mm with a scan time of about 5 min. Fifty images (z-direction) with no interslice gap were obtained for each subject. Muscle volume and mean cross-sectional area (CSA) of the m. quadriceps femoris was analyzed from the first image not displaying m. gluteus maximus and ending with the last image in which m. rectus femoris appeared^19^. Within this segment (10–18 cm of the thigh), every third image was assessed by manual planimetry using imaging software (Image J, National Institutes of Health, Bethesda, MD). The average of 2 measures showing less than 1% difference between values was multiplied by slice thickness to obtain muscle volume. Although pre- and post-images were analyzed in parallel, the individual who performed the analysis was blind with regard to the time-point of the images.

**Figure 2:**
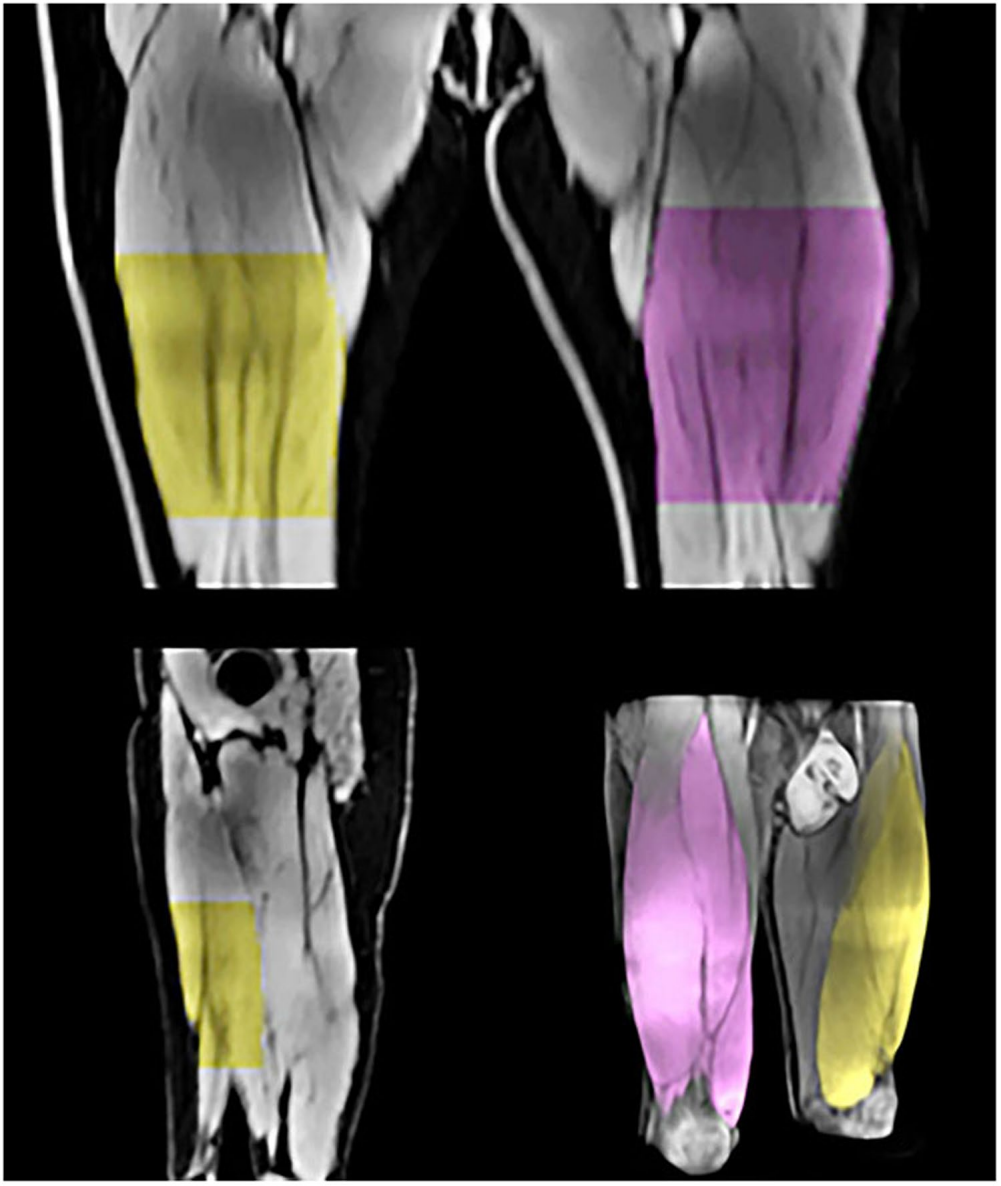
Illustration of the automatic detection of the targeted partial muscle volume, as well as the whole quadriceps femoris volume (bottom right). The partial volume was calculated between points-of-interests (proximal and distal start/stop image) that were placed in the first slice not showing the gluteus maximus muscle and the last slice still including the rectus femoris on both sides as described in the manual analysis (see text for further details). The upper panel displays the detected partial region of the quadriceps in the frontal plane (anterior view). The bottom left panel display the detected region in the sagittal plane.

The images for the automatic segmentation were acquired in the same session as the ones used for the manual analysis (i.e., the subject did not move in between the two image acquisitions). A standard T1-weighted gradient echo sequence with a 2-point Dixon reconstruction (Dixon VIBE) was acquired in the axial plane from the top of the femur to below the knees as two separate image-stacks. Acquisition parameters were TE1 2.4 ms, TE2 4.7, TR 6.7 ms, FOV 50 × 35 cm, voxel size 2.2 × 2.2 × 3.5 mm with a scan time of 22–32 s per image stack. Water, fat, in-phase and out-of-phase images were calculated at the scanner. All datasets were exported to AMRA^®^ Researcher where quantification of the fat-free muscle volume and the proportion of intra-muscular fat of the bilateral m. quadriceps using a non-rigid multi-atlas segmentation technique was performed^13,14,16^. A manual quality assurance step was thereafter applied where five datasets failed due to image distortion. This was because the increase in size in the feet-head direction that was needed to cover the relevant anatomy (42–48 cm) resulted in geometrical distortions making the process of merging the different image volumes together problematic, and as a consequence jeopardizing the volume calculations. To be able to compare the muscle volumes from the automatic and manual segmentation, a restricted portion of the automatic volume was calculated between points-of-interest that were placed in the first slice not showing the gluteus maximus muscle and the last slice still including the rectus femoris on both sides as described for the manual analysis (called partial volume). This was done in addition to the full volume analysis of the quadriceps muscle calculated by the automatic method (Fig. 2).

### Data analysis

After assessing data distribution through histograms and the Shapiro-Wilk test, it was deemed that parametric testing was appropriate. The relationship between the manual and automated method of muscle volume measurements was assessed using linear regression (Pearson’s r-value). Further, the systematic and random error (mean bias and standard deviation (SD) of bias) was assessed using Bland-Altman plots. The difference in mean values between the two methods (systematic bias) and the sensitivity of the methods to detect changes pre- to post-intervention (averaged across legs) was assessed using paired t-tests. An alpha-level of 5% was accepted as significant for all statistical analyses (P < 0.05). All of the above variables were computed in GraphPad Prism (GraphPad Software Inc, California, USA). In addition, the typical error of the estimate (expressed in %) was computed using the spreadsheets provided at sportsci.org^20^, and the intra-class correlation (ICC) using R version 3.5.1. Data are expressed as means ± SD unless otherwise stated.

## Results

**Figure 3:**
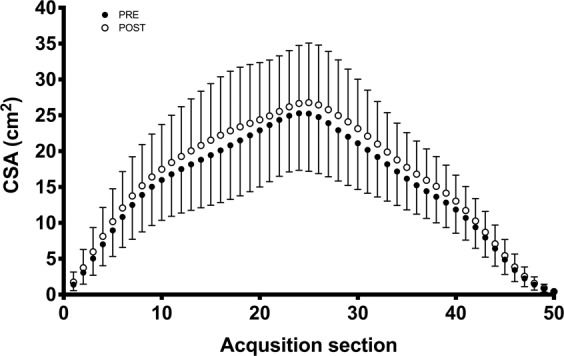
Training-induced changes in whole quadriceps volume measured with the automated method only. Each data-point (1–50) represents an acquisition section of the muscle. The acquisition sections have been computed by dividing each subject’s quadriceps muscle into 50 sections ranging from the first to the last image where the quadriceps muscle was detected. This means that, in this figure, taller subjects (longer thighs) have thinner sections and vice versa. Data are means with SD.

Muscle volume of the complete m. quadriceps, measured with the automated technique, was 2056.7 ± 617.7 cm3 before training and 2152.2 ± 615.1 cm3 after training. Thus, whole-quadriceps volume increased 5.1 ± 5.5% with training (Fig. 3, P < 0.0001). The fat volume was 55.5 ± 18.0 cm^3^ before training compared with 54.2 ± 17.4 cm3 after training (P > 0.05). The amount of muscle fat infiltration was 4.1 ± 0.9% before training and 3.9 ± 0.8% after training (P < 0.001).

In the targeted region of the quadriceps (partial volume), which was assessed with both methods, muscle volume measured with the manual method showed a 6.0 ± 5.0% increase with training (from 925.7 ± 303.2 cm3 to 980.9 ± 312.5 cm3). The corresponding increase with the automated method was 4.8 ± 8.3% (from 1022.1 ± 339.6 cm3 to 1071.2 ± 340.3 cm3). Both methods were able to detect a significant increase in muscle volume with the training intervention (P < 0.0001), and the magnitude of hypertrophy was similar across methods (P > 0.05).

**Figure 4:**
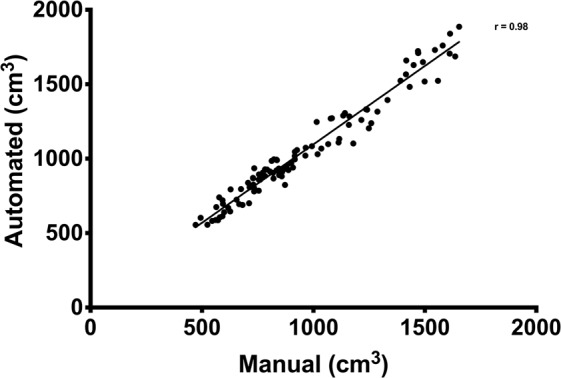
Correlation between the automated and manual segmentation technique for assessing partial muscle volume of the quadriceps muscle. Each data point represents one leg at one time-point measured with both methods.

**Figure 5:**
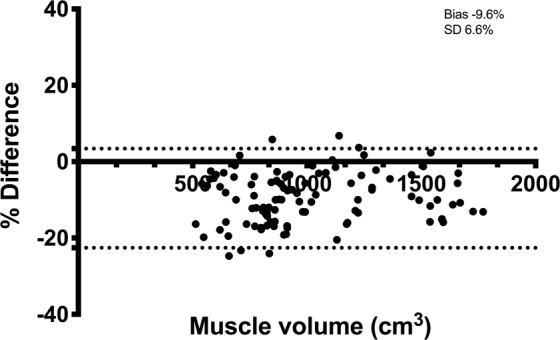
Bland-Altman plot of quadriceps volume determined with the automated and manual segmentation technique. Quadriceps volume on the x-axis and the difference between the methods as a percentage on the y-axis. Dotted lines represent 95% limits of agreement. Bias and SD of bias are expressed in %.

There was a very strong correlation (r = 0.98, 95% confidence interval (CI): 0.97–0.99, P < 0.0001) between manual and automated assessment of the partial quadriceps volume (Fig. 4). The 95% limit of agreement (Fig. 5) was −93.3 ± 137.8 cm3 (−9.6 ± 13.0%). The ICC was 0.98 and the random error 6.7%. Specifically, the ICC for consistency was 0.98 and the ICC for agreement was 0.94. There was a significant systematic bias between the two methods where partial quadriceps volume measured with the automated method was 9.6% greater than with manual segmentation (P < 0.0001).

## Discussion

The importance of muscle mass for human health, performance and functional capacity underscores the need for accurate and time-efficient assessment methods. Accordingly, the goal of this study was to validate an MRI-based automated technique for assessment of muscle volume that can be applied in future large-scale studies where specific muscle volumes are a primary outcome variable. More specifically, we addressed the agreement between automated and manual segmentation of a targeted region of the quadriceps muscle and explored the sensitivity of the automated technique to detect small but meaningful changes in muscle size consequent to 8 weeks of resistance training in young healthy individuals. Our results showed excellent agreement between the manual and automated method, and both methods were able to detect small but meaningful training-induced changes in muscle volume. Given that the automated method is unbiased, time-effective and can be used to determine fat- and fat-free muscle volumes of the entire body, we propose that it could be a viable option in future wider-scale studies.

The excellent correlation between the two segmentation methods was comparable with previous reports using similar automated techniques, but on more gross muscle volumes^13^. However, as indicated by the Bland-Altman graphs and lower agreement than consistency in ICC, there was a systematic difference between the two methods when comparing absolute values of the partial quadriceps volume. Thus, the methods are not necessarily interchangeable with each other, and caution is warranted when used in cross-sectional studies where the absolute value might be of interest. This absolute difference is, however, of less importance in longitudinal studies where the change in muscle volume is the main outcome variable. For these purposes, the time-efficient automated method is a reliable option.

The consistent bias in absolute values is perhaps not surprising since the two methods rely on different assumptions, image acquisition sequences and settings, reconstruction algorithms and segmentation techniques. The manual segmentation is influenced by anatomical skills of the operator, as well as choices made when tracing the region of interest. This applies not only to the actual tracing of the muscle, but also to the judgement and selection of the start and stop image for manual analysis in the targeted region. As tracing decisions are automated and unbiased with the automated technique, there is very limited test-retest variation^13^, and no inter-operator variability or need for an experienced reader performing quality control^21^. This underscores some of the advantages with the automated method and in fact, ad hoc examination of the segmented images revealed that a large part of the systemic bias could be explained by differences in where the segmentation edge was placed. The manual operator was careful not to include fat in the muscle volume being traced and therefore placed the segmentation line inside the muscle tissue area rather than directly on the muscle fascia/subcutaneous fat area. In contrast, the automated segmentation technique includes all voxels containing <50% fat in the “fat-free” muscle volume. This results in a more generous segmentation edge for the automated compared with the manual analysis (i.e. closer to the subcutaneous fat). While we selected a specific region of the quadriceps to allow for comparison with existing manual methods^22,23^, it should be noted that the automated AMRA-technique, as reported in the current study, also records the volume of the entire knee extensor group^13^. Thus, selecting a specific region of interest within the quadriceps muscle, as we typically have done in past studies to save time and to be able to trace all muscle borders reliably, is not necessary with the automated technique.

An important outcome of this study was the confirmation that the automated method was sensitive enough to detect meaningful changes in muscle volume following a training intervention. Being able to accurately assess compartmental changes in body composition is important in sports science settings to assess the efficacy of training interventions, but also in weight-sensitive sports where athletes use extreme methods to reduce weight or fat mass in order to gain competitive advantages. Exercise interventions employing knee-extensor resistance training report an average hypertrophy of 0.14% per day^24^. Thus, a typical 6–8-week intervention should result in hypertrophic magnitudes of 5–7%. The change in muscle volume assessed by the automated method was within this range, and the partial-volume analysis was also comparable to the established manual method, albeit with a slightly greater variability in the percentage hypertrophy values. The ability to accurately and reliably detect changes in muscle volume in response to training is also important given that time-saving manual strategies, such as using only a single slice CSA as a proxy of hypertrophy, is only moderately well correlated with muscle volume^25^, and does not account for regional differences in hypertrophy along the muscle length^26^.

Apart from the major manpower and time advantage, the use of this particular automated technique is independent of field strength and image resolution as long as a specific MRI sequence is used^14^. Thus, given that the analysis is not limited to a specific MRI scanner, it is possible to use this method in almost any facility offering MRI scans. This opens up for rapid and fully comparable volume quantifications in, for example, large-scale multi-center studies. One methodological aspect that should be recognized, however, is that the 2-point Dixon acquisition, relying on intensity corrected water/fat-only images, will exclude small volumes of intramuscular fat which likely would be included in the manual analysis. In the current study, the intramuscular fat proportion amounted to 4% of the measured muscle volume, where voxels with fat content higher than 50% were excluded from the muscle component^14^. While the exclusion of fat voxels should be considered an advantage with the automated method, it could limit direct comparisons of absolute values to previously published data, in particular data on muscles that are fattier than the knee extensors, or in obese individuals. For clinical purposes, however, it is important to highlight that the present automated method is compatible with compartmental fat quantification based on the same fat-water separated images. Thus, fat- and fat-free mass can be traced in parallel within the regions of interest, and the volumes of total adipose tissue as well as visceral and subcutaneous adipose fat tissue can be quantified^27,28^. This approach could be very valuable in clinical settings, such as studies on sarcopenia, neurological diseases, obesity and myopathies, where several body compositional measures are of interest, including muscle fat infiltration^29–33^. In addition to the observed increase in muscle volume, the automated method also showed a significant decrease in muscle fat infiltration following the training intervention. Muscle fat infiltration has been shown to be related to functional outcomes, both in neuromuscular disorders as well as in sarcopenia-related research^32,34^.

To cover the region from the top of the caput femoris to below the knee, the slab sizes were adjusted by the technician performing the scan to ensure proper coverage of the whole upper leg, resulting in slab sizes of 30–35 cm for the automated MRI acquisition. Unfortunately, in five individuals, the increase in size in the feet-head direction that was needed to cover the relevant anatomy resulted in geometrical distortions. These data sets were therefore excluded from further automated analysis. For future studies it is important to stress that to ensure proper coverage without geometrical distortions, it is preferred to not exceed 30–35 cm in the feet-head direction and instead add more image stacks to cover the anatomy of interest.

In conclusion, we have validated a rapid and automated method for quantification of muscle-specific volumes. The automated method showed near perfect correlation with manual segmentation and, equally important, was able to detect small but relevant increases in muscle hypertrophy after 8 weeks of resistance training in healthy adults. Given that the automated technique also allows for quantification of intra- and extra-muscular fat volumes, we believe this method could have broad application in clinical and sport settings where there is a pressing need for accurate, reproducible and time-efficient means to monitor changes in body composition and muscle mass.

## Data Availability

All original data are available at request from the corresponding author.
